# Determinants of very low birth weight in India: The National Family Health Survey – 4

**DOI:** 10.12688/wellcomeopenres.17463.1

**Published:** 2022-01-24

**Authors:** Liss Scaria, Biju Soman, Babu George, Zulfikar Ahamed, Sankar Hariharan, Panniyammakal Jeemon

**Affiliations:** 1 AMCHSS, Sree Chitra Thirunal Institute for Medical Sciences and Technology, Trivandrum, Kerala, 695011, India; 2 Child Development Centre, Government Medical College, Trivandrum, Kerala, 695011, India; 3 Pediatrics, Government Medical College, SAT Hospital, Trivandrum, Kerala, 695011, India

**Keywords:** Low birth weight, very low birth weight, determinants, National Family Health Survey, India

## Abstract

Background

Low birth weight (LBW) is susceptible to neonatal complications, chronic medical conditions, and neurodevelopmental disabilities. We aim to describe the determinants of very low birth weight (VLBW) in India based on the National Family Health Survey – 4 (NHFS-4).

Methods

Data from the NFHS 4 on birthweight and other socio-demographic characteristics for the youngest child born in the family during the five years preceding the survey were used. Data of 147,762 infant–mother pairs were included. Multiple logistic regression models were employed to delineate the independent predictors of VLBW (birth weight<1500 g) or LBW (birth weight <2500 g).

Results

Of the 147,762 children included in the study, VLBW and LBW were observed in 1.2% and 15.8% of children, respectively. The odds of VLBW were higher in female children (aOR: 1.36, 95% CI: 1.15–1.60), among mothers aged 13–19 years (aOR: 1.58, 95% CI: 1.22–2.07), mothers with severe or moderate anaemia (aOR: 1.61, 95% CI: 1.34–1.94), mothers without recommended antenatal care (aOR: 1.47, 95% CI: 1.31–1.90), maternal height less than 150 cm (aOR: 1.54, 95% CI: 1.29–1.85) and among mothers with multiple pregnancy (aOR: 21.34, 95% CI: 14.70–30.96) in comparison to their corresponding counterparts. In addition to the variables associated with VLBW, educational status of mothers (no education; aOR: 1.08, 95% CI: 1.02–1.15 and primary education; aOR: 1.16, 95% CI: 1.08–1.25), caste of the children (scheduled tribe; aOR: 1.13, 95% CI: 1.03–1.24), and wealthiness of the family (poorest wealth quintiles; aOR: 1.11, 95% CI: 1.03–1.19) were associated with LBW.

Conclusions

Interventions targeting improvements in antenatal care access, maternal health, and nutritional status may reduce the number of VLBW infants. Social determinants of LBW require further detailed study to understand the high propensity of low birth-weight phenotypes in the disadvantaged communities in India.

## Introduction

Low birth weight (LBW), defined as birth weight less than 2500 g, is a significant public health problem globally, and remains as a major health issue in India ^1, 2^. Very low birth weight (VLBW), a sub-group with birth weight <1500 g, is a high-risk group with considerable mortality and morbidity ^3– 5^. Advances in medical care, treatment facilities, and progress in availability of these services over the last several decades including the establishment of level three nurseries for neonates across India, improved the survival of both LBW and VLBW babies ^6– 8^. However, the survived babies with LBW are susceptible to neonatal complications, recurrent hospitalisations, chronic medical conditions and neurodevelopmental disabilities like intellectual disabilities, and learning disabilities ^9, 10^. It also increases the future risk of chronic diseases and other co-morbidities. For example, adult diseases such as hypertension, dyslipidaemia and insulin resistance are closely related to a LBW, leading to markedly increased rates of cardiovascular, metabolic and renal diseases in later life ^11^. Understanding the determinants of VLBW among infants is critical for planning further interventions in reducing the associated morbidity and mortality. We describe the socio economic and maternal determinants of VLBW infants in India based on the National Family Health Survey – 4 (NHFS-4) data.

## Methods

### Ethical statement

Our study is based on a secondary analysis of existing data from NFHS-4, which is an anonymous and publicly available dataset. The dataset has no identifiers of the survey participants. At the beginning of the survey, the interviewer took informed consent from each participant after explaining the purpose of the study. The informed consent explained that the participation was voluntary, and participants had the right to refuse or stop the interview at any point. The NFHS-4 obtained ethical clearance from the Ethical Review Board of the International Institute for Population Science (IIPS), which performed these surveys. We registered at the DHS site as data users and submitted a research proposal to study the determinants of VLBW. The Demographic Health Survey (DHS) program gave access to the data after reviewing the submitted proposal ( 10.6084/m9.figshare.16606787). We downloaded the required data from https://www.dhsprogram.com/data/available-datasets.cfm. We accepted all terms and conditions attached with the data sharing policy of DHS.

### Data source

We used data from the NFHS 4 which was conducted during the year 2015–2016. The first NHFS survey began during early 1990s. The NFHS presents nationally representative data on population, health, and nutrition for India including its states as well as union territories. The survey also intended to offer state and national-level estimates of fertility, mortality, family planning, adolescent reproductive health, high-risk sexual behavior, HIV-related knowledge and use of healthcare services in the country.

Using a multi-stage sample design, NFHS 4 covered sample households all over India. A stratified two-stage sampling design was adopted for the NFHS-4 survey. For all districts surveyed, a uniform sampling design was used considering rural and urban areas as strata. To select primary sampling units (PSUs), the Census of India 2011 served as the sampling frame. The PSUs in rural areas were villages whereas it was census enumeration blocks (CEBs) in urban areas. The villages and CEBs were selected from the sampling frame with probability proportional to size (PPS) sampling. A household mapping and listing operation was performed at every selected PSU before the main survey. In the second stage, a random selection of 22 households from each PSU was done. The details on study design, sampling, and data collection schedule of the NFHS have been published elsewhere ( http://rchiips.org/Nfhs/NFHS-3%20Data/VOL-1/India_volume_I_corrected_17oct08.pdf, https://dhsprogram.com/pubs/pdf/FR339/FR339.pdf). The fourth round (NFHS-4) collected data from 30 states and six union territories from India. The NFHS-4 survey gathered information from 699,686 women, and 112,122 men.

### Study participants

**Figure 1.**
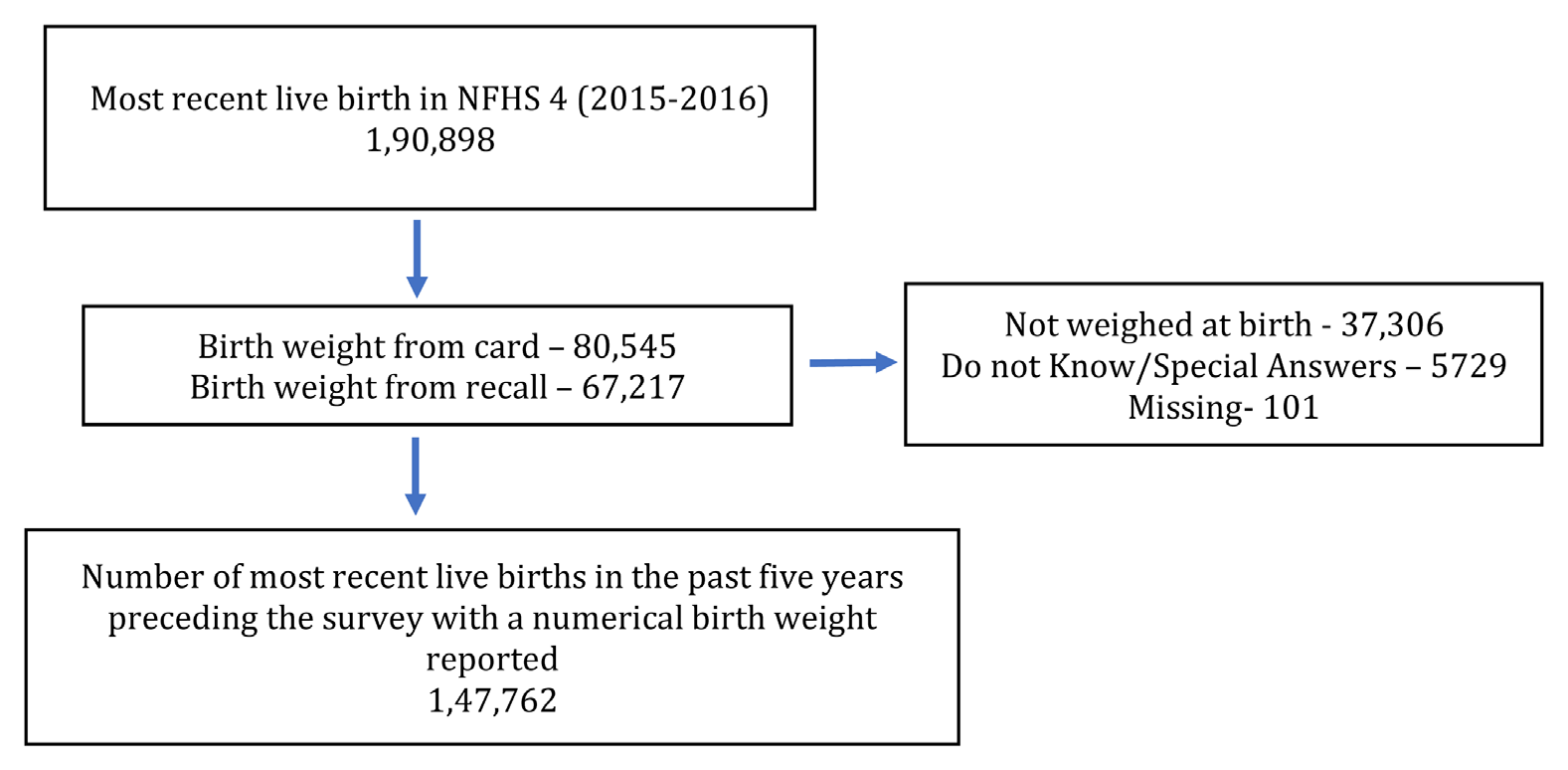
Flow chart description detailing study inclusion.

We used the data on birthweight and other socio-demographic characteristics for the youngest child born in the family during the past five years preceding the survey (n=190,898 children). Data of 37,306 children were reported as not weighed. Additionally, data from 5729 children were reported as special answers or do not know ( Figure 1). Children with a birth weight of less than 1500 g and birth weight between 1500–2499 g were considered as VLBW and LBW, respectively. We included 147,762 infant-mother pairs meeting the inclusion criteria in the study ( Figure 1).

### Study variables

**Figure 2.**
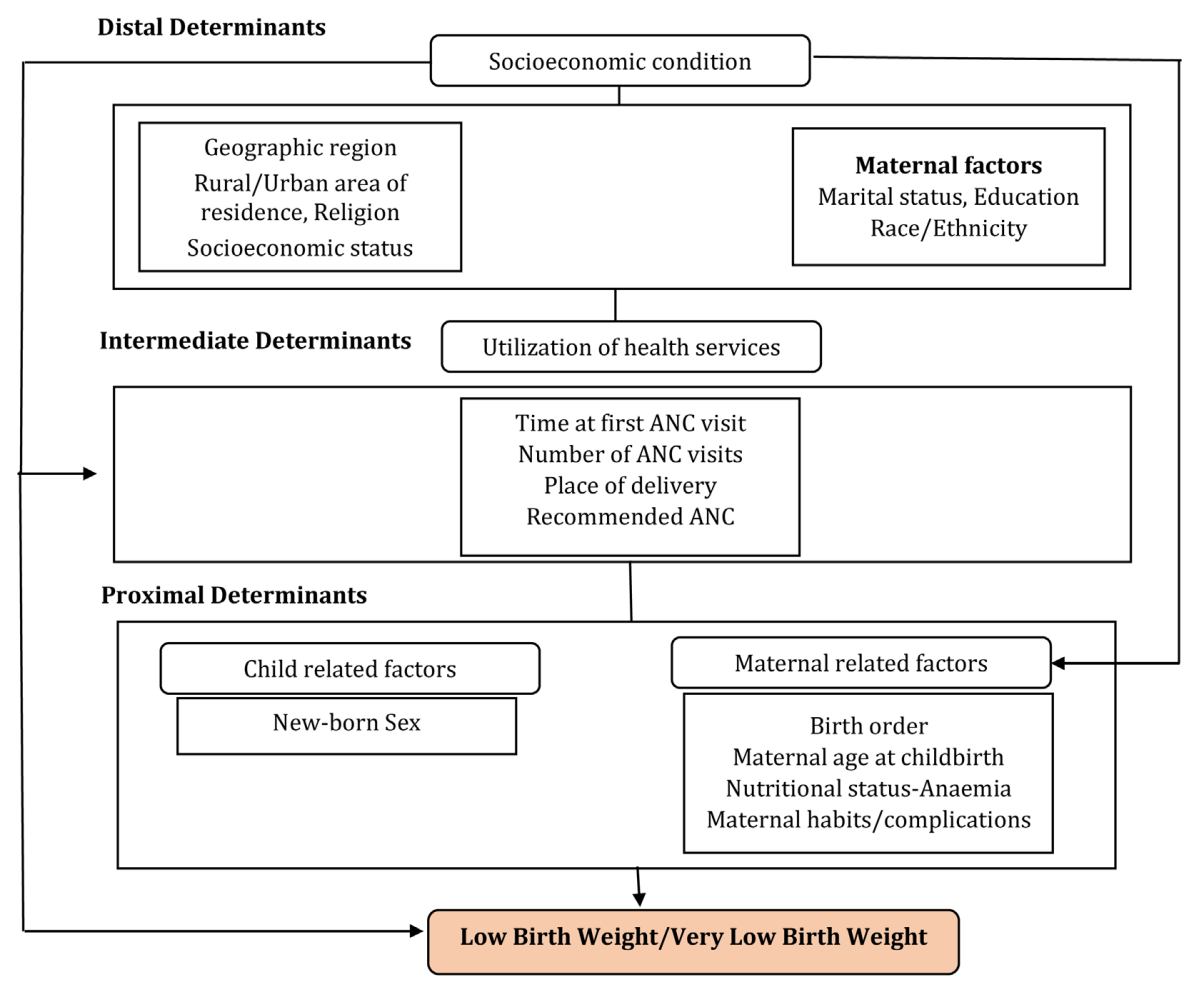
Conceptual hierarchy-based model ANC=Antenatal Care used to analyze factors associated with low birth weight. (Adapted from Falcão *et al.* BMC Pregnancy and Childbirth 2020)

We grouped the study variables into three blocks representing distal, intermediate and proximal determinants, using a conceptual hierarchy-based approach ^12^ *i.e.*, socioeconomic characteristics, use of the healthcare services or the programmatic factors including antenatal care (ANC), and maternal and new-born characteristics, respectively ( Figure 2).

The key study variables were individual and household socio-demographic characteristics including age and education of the mother, wealth index, marital status, religious background, and place of residence ( Table 1). Reproductive characteristics of the mother included age at birth of the index child, birth order, birth interval, the type of complications during pregnancy and general health behaviours including smoking and alcohol status. The antenatal check-up (ANC) status included the timing of the first ANC visit, number of ANC visits, tetanus injection during pregnancy, place of delivery, and service accessibility. Anthropometric measures included height and body mass index of the mother. We also included the anaemia status of the mother as a study variable.

**Table 1.** List of explanatory variables and their categories used in this study. OBC- Other Backward Class, ANC- Antenatal Care, BMI- Body Mass Index.

Maternal or child related factors	
Sex of the child	Male, female
Birth order of the child	1-3, 4 or more
Mother's age at birth in years	<19, 20-34, 35-49
Anaemia	Moderate/severe anaemia, Mild Anaemia, Not anaemic Mild anaemia (10.0-10.9 grams/decilitre for pregnant women), moderate anaemia (7.0-9.9 g/dl), and severe anaemia (less than 7.0 g/dl)
Thyroid Disease	Yes, No (self-reported)
Smoking	Yes, no (current smoking status)
Alcohol consumption	Yes, No (current alcohol consumption)
Type of delivery	Normal Delivery, Caesarean
Pregnancy type	Singleton, Multiple
Duration of pregnancy	<9 months, > 9 months
Mother ‘s Height	Height <150 cm, Height >150 cm
BMI of the mother	>18.5, <18.5 (calculated using height and weight of mother)
**Programmatic factors**	
Time at first ANC visit	1st trimester, after 1st trimester
Number of ANC visits	<4 ANC visits, >4 visits
Place of delivery	Institutional, Home
ANC – recommended	ANC in first trimester, at least four antenatal visits, at least one tetanus toxoid (TT) injection and iron folic acid tablets or syrup taken for 100 or more days
**Socio-economic factors**	
Area of residence	Urban, rural
Social group of mothers	Scheduled caste, Scheduled tribe, OBC, Others
Mother's schooling	No education, Primary, Secondary or Higher
Wealth index of the household	Poorest/poorer, middle, richer/richest (Based on scores on ownership of consumer goods and household characteristics: https://dhsprogram.com/pubs/pdf/FR339/FR339.pdf)
Religion	Hindu, Christian, Muslim, Others
Marital Status	Currently married, Not married currently
**Geographic region**	
Northern states	Chandigarh, Delhi, Haryana, Himachal Pradesh, Jammu & Kashmir, Punjab, Rajasthan, Uttarakhand
Central states	Chhattisgarh, Madhya Pradesh, Uttar Pradesh
Eastern states	Bihar, Jharkhand, Odisha, West Bengal
North Eastern states	Arunachal Pradesh, Manipur, Meghalaya, Mizoram, Nagaland, Sikkim, and Tripura
Western states	Dadra & Nagar Haveli, Daman & Diu, Goa, Gujarat, Maharashtra
Southern States	Andaman & Nicobar Islands, Andhra Pradesh, Karnataka, Kerala, Lakshadweep, Puducherry, Tamil Nadu, Telangana

### Data analysis

We used STATA Version 16.1 STATA Corp (RRID:SCR_012763) for the data analysis. We explored the bivariate associations between socio-demographic and maternal variables and low birth-weight phenotypes (VLBW and LBW). The statistically significant predictors ( *P*<0.10) from the bivariate model were further analysed using multiple logistic regression models to establish the independent association between these variables and LBW phenotypes. A correlation matrix was employed to check multicollinearity. In the final multivariable regression model, we excluded BMI, type of delivery, place of delivery, pregnancy duration, first ANC visit and religion to avoid multicollinearity. We generated adjusted odds ratio (aOR) with their 95% confidence intervals (CI). 

## Results

### General characteristics

Of the 147,762 children included in the study, 1722 (1.2%) were with VLBW. In total 23,308 (15.8%) children had LBW. More than half (54.5%) of the children were boys ( Table 2). Nearly two-thirds (64%) of the mothers reported height greater than 150 cm. The body mass index was more than 18.5 kg/m ^2^ in four-fifths (80.0%) of the mothers. 87% of the mothers belonged to the 20–34 years at the time of childbirth. About 19% each belonged to scheduled caste and scheduled tribes. Nearly two-thirds of the mothers (64.8%) reported secondary or higher education. 40% belonged to poorer or poorest wealth quintiles. One-third of the mothers reported severe or moderate anaemia. More than two-thirds (72.4%) of the mothers reported their first antenatal care (ANC) visits during the first trimester itself.

**Table 2.** Sample distribution by selected background characteristics – NFHS-4.

	LBW, n (%)	VLBW, n (%)	Normal Weight, n (%)	Total n (%)
**Gender**				
Male	11870(14.74)	872(1.08)	67764(84.17)	80506(100)
Female	11438(17.01)	850(1.26)	54968(81.73)	67256(100)
**Birth Order**				
1 to 3	20236(15.70)	1472(1.14)	107220(83.16)	128928(100)
>4	3072(16.31)	250(1.33)	15512(82.36)	18834(100)
**Age of mother**				
13–19 years	2058(10.16)	9101(44.92)	9101(44.92)	20260(100)
20–34 years	19306(15.34)	1250(0.99)	105301(83.67)	125857(100)
>35 years	1045(14.26)	80(1.09)	6201(84.64)	7326(100)
**Marital Status**				
Currently married	389(10.00)	1478(37.99)	2023(52.01)	3890(100)
Currently not married	22919(15.95)	20(0.01)	120709(84.03)	143648(100)
**Place of residence**				
Urban	6376(15.21)	500(1.19)	35033(83.59)	41909(100)
Rural	16932(16.00)	1222(1.15)	87699(82.85)	105853(100)
**Social Group**				
SC	4788(17.69)	381(1.41)	21902(80.91)	27071(100)
ST	3717(13.96)	193(0.72)	22716(85.32)	26626(100)
OBC	9382(16.09)	718(1.23)	48209(82.68)	58309(100)
Others	4371(15.14)	344(1.19)	24164(83.67)	28879(100)
**Educational status**				
No education	5835(17.91)	489(1.50)	26257(80.59)	32581(100)
Primary	3438(17.64)	254(1.30)	15793(81.05)	19485(100)
Secondary/Higher	14035(14.67)	979(1.02)	80682(84.31)	95696(100)
**Wealth Quintiles**				
Poorest/Poorer	10163(17.1)	788(1.33)	48472(81.57)	59423(100)
Middle	4961(15.69)	336(1.06)	26322(83.25)	31619(100)
Richer/Richest	8184(14.43)	598(1.05)	47938(84.52)	56720(100)
**Religion**				
Hindu	18474(16.64)	1336(1.20)	91184(82.15)	110994(100)
Muslim	3088(15.46)	282(1.41)	16609(83.13)	19979(100)
Christian	876(8.57)	37(0.36)	9304(91.06)	10217(100)
Others	870(13.24)	67(1.02)	5635(85.74)	6572(100)
**Geographic region**			
Northern states	5151(17.67)	411(1.41)	23587(80.92)	29149(100)
Central states	6550(18.03)	588(1.62)	29198(80.36)	36336(100)
Eastern states	4406(14.92)	255(0.86)	24868(84.22)	29529(100)
North Eastern states	1994(9.78)	117(0.57)	18287(89.65)	20398(100)
Western states	2274(17.48)	170(1.31)	10567(81.22)	13011(100)
Southern States	2933(15.17)	181(0.94)	16225(83.90)	19339(100)
**Anaemia**				
Severe/Moderate	6634(18.11)	512(1.4)	29483(80.49)	36629(100)
Mild	4705(14.64)	316(0.98)	27117(84.38)	32138(100)
Not Anaemic	6806(13.81)	408(0.83)	42074(85.36)	49288(100)
**Tobacco use**				
User	1876(13.92)	118(0.88)	11485(85.21)	13479(100)
Non-user	21432(15.96)	1604(1.19)	111247(82.85)	134283(100)
**Alcohol**				
No	22973(15.82)	1705(1.17)	120566(83.01)	145244(100)
Yes	335(13.3)	17(0.68)	2166(86.02)	2518(100)
**Thyroid Disease**				
No	22797(15.75)	1674(1.16)	120294(83.1)	144765(100)
Yes	355(15.67)	35(1.55)	1875(82.78)	2265(100)
**First ANC visit**				
During 1 ^st^ Trimester	14370(15.15)	1049(1.11)	79445(83.75)	94864(100)
After 1 ^st^ Trimester	5855(16.17)	404(1.12)	29941(82.71)	36200(100)
**Recommended ANC care**				
Appropriate	18711(16.56)	1435(1.27)	92829(82.17)	112975(100)
Inappropriate	4597(13.21)	287(0.83)	29903(85.96)	34787(100)
**Place of delivery**				
Home delivery	1964(17.58)	160(1.43)	9049(80.99)	11173(100)
Institutional	21344(15.63)	1562(1.14)	113683(83.23)	136589(100)
**Pregnancy type**				
Singleton	22584(15.43)	1556(1.06)	122208(83.51)	146348(100)
Multiple pregnancy	724(51.2)	166(11.74)	524(37.06)	1414(100)
**Pregnancy duration**				
Preterm	2459(25.97)	538(5.68)	6472(68.35)	9469(100)
Full term	20849(15.08)	1184(0.86)	116260(84.07)	138293(100)
**Height**				
<150 cm	9384(18.03)	718(1.38)	41958(80.60)	52060(100)
> 150 cm	13610(14.51)	977(1.04)	79242(84.45)	93829(100)
**BMI**				
Underweight	6278(19.53)	466(1.45)	25404(79.02)	32148(100)
Normal Weight	13544(15.14)	972(1.09)	74926(83.77)	89442(100)
Overweight	3168(13.05)	256(1.05)	20850(85.89)	24274(100)

SC- Scheduled Caste, ST- Scheduled Tribe, OBC- Other Backward Class, ANC- Antenatal Care, BMI- Body Mass Index

### Factors associated with very low birth weight

In the bivariate analysis, the child’s gender, height, BMI, birth order, age of the mother, anaemia level, tobacco and alcohol use, thyroid disease, antenatal visits, place of delivery, multiple pregnancy, caste, religion, educational status, wealth quintile, geographic region, and pregnancy duration, were associated with VLBW (Supplementary Table 1: 10.6084/m9.figshare.18393749). In the multivariable logistic regression model, odds of VLBW were higher in female children when compared with male children (aOR: 1.36, 95% CI: 1.15–1.60) ( Table 3). Mothers aged 13–19 years had higher odds for VLBW when compared with mothers aged 20–34 years (aOR: 1.58, 95% CI: 1.22–2.07). Children from Eastern states had lower odds for VLBW (aOR: 0.47, 95% CI: 0.33–0.67) as compared with children from Western states. The odds of VLBW were 1.61-times higher in mothers with severe or moderate anaemia *versus* non-anaemic mothers (aOR: 1.61, 95% CI: 1.34–1.94). Mothers who did not follow recommended ANC had 47% higher odds of VLBW compared to the reference group of mothers who adhered to ANC recommendations (aOR: 1.47, 95% CI: 1.31–1.90). The odds of having VLBW was 21-times higher (aOR: 21.34, 95% CI: 14.70–30.96) among children of mothers with the multiple pregnancy *versus* singleton pregnancy. Mothers whose height was less than 150 cm had 54% higher odds of VLBW compared to mothers with height greater than 150 cm (aOR: 1.54, 95% CI: 1.29–1.85).

**Table 3.** Logistic regression of the selected characteristics with birth weight <1500gms as outcome compared with normal birth weight - Model 1.

		VLBW-Normal Birth Weight, (n= 94,705)	
	Prevalence of VLBW (%)	Odds Ratio	95% Conf. Interval
**Gender**			
Male	1.27	Ref	
Female	1.52	1.36 ***	1.15 to 1.60
**Birth order**			
One to three	1.35	Ref	
>4	1.59	0.8	0.63 to 1.01
**Age of the mother**		
13–19 years	49.54	1.58 **	1.22 to 2.07
20–34 years	1.19	Ref	
>35 years	1.29	1.3	0.92 to 1.86
**Education**			
No education	1.86	1.24	0.99 to 1.55
Primary	1.61	0.95	0.75 to 1.20
Secondary	1.21	Ref	
**Social Group**			
SC	1.74	1.05	0.79 to 1.39
ST	0.85	0.81	0.53 to 1.17
OBC	1.49	0.96	0.76 to 1.22
Others	1.42	Ref	
**Wealth index**			
Poorest/Poorer	1.63	1.18	0.92 to 1.51
Middle	1.28	0.87	0.68 to 1.11
Rich/Richest	1.25	Ref	
**Geographic region**			
Northern states	1.74	1.01	0.73 to 1.42
Central states	2.01	1.05	0.78 to 1.42
Eastern states	1.03	0.47 ***	0.33 to 0.67
North Eastern States	0.64	0.77	0.50 to 1.18
Western states	1.61	Ref	
Southern States	1.12	0.71	0.49 to 1.03
**Anaemia**			
Severe/Moderate	1.71	1.61 ***	1.34 to 1.94
Mild	1.15	1.3	0.92 to 1.54
Not anaemic	0.96	Ref	
**Tobacco Use**			
Users	1.02	1.18	0.84 to 1.65
Non-user	1.42	Ref	
**Alcohol drinking**			
Yes	1.39	0.81	0.33 to 1.95
No	0.78	Ref	
**Recommended ANC ^a^**			
No	1.55	1.47 **	1.31 to 1.90
Yes	0.96	Ref	
**Pregnancy type**			
Multiple	1.26	21.34 ***	14.70 to 30.96
Singleton	24.06	Ref	
**Height**			
<150 cm	1.68	1.54 ***	1.29 to 1.85
> 150 cm	1.22	Ref	

*p<.05 **p<.01 ***p<.001 ^a^ANC in first trimester, at least four antenatal visits, at least one tetanus toxoid (TT) injection and iron folic acid tablets or syrup taken for 100 or more days. SC- Scheduled Caste, ST- Scheduled Tribe, OBC- Other Backward Class, ANC- Antenatal Care, BMI- Body Mass Index

### Factors associated with low birth weight

In the bi-variate analysis, the child’s gender, height, BMI, birth order, age of the mother, anaemia level, tobacco and alcohol use, antenatal visits, place of delivery, multiple pregnancy, place of residence, caste, religion, educational status, wealth quintile, geographic region, timing of first ANC visits and appropriate ANC use were associated with LBW (Supplementary Table 2: 10.6084/m9.figshare.18393758). In the multivariable logistic regression model ( Table 4), odds of LBW were higher in girl children when compared to boys (aOR: 1.21, 95% CI: 1.15–1.26). Children with birth order greater than four were having lower odds for LBW than children with birth order one to three (aOR: 0.86, 95% CI: 0.80–0.92). Mothers aged 13-19 years had higher odds for VLBW when compared to mothers aged 20–24 years (aOR: 1.17, 95% CI: 1.06–1.26). Mothers with no education (aOR: 1.08, 95% CI: 1.02–1.15) and those with primary education (aOR: 1.16, 95% CI: 1.08–1.25) had higher odds of LBW as compared to those in the secondary education category. Children who belonged to scheduled tribe had 1.13-times higher odds for LBW *versus* children from other forward caste (aOR: 1.13, 95% CI: 1.03–1.24). Children from poorest/poorer wealth quintiles had higher odds of LBW *versus* those from rich or richest wealth quintiles (aOR: 1.11, 95% CI: 1.03–1.19). When compared with children from Western states, those from Eastern states (aOR: 0.75, 95% CI: 0.68–0.82), North-Eastern states (aOR: 0.61, 95% CI: 0.55–0.69) and Southern states (aOR: 0.90, 95% CI: 0.82–0.99) had lower odds for LBW. The odds of LBW were 1.20-times higher in mothers with severe or moderate anaemia *versus* non-anaemic mothers (95% CI: 1.13–1.26). Mothers who followed recommended ANC had lower odds of LBW compared with the reference group of mothers who did not follow ANC recommendations (aOR: 0.78, 95% CI: 0.73–0.83). The odds of having LBW were eight-times higher (aOR: 8.68, 95% CI: 7.05–10.68) among children of mothers with the multiple pregnancy *versus* singleton pregnancy. Mothers whose height was less than 150 cm had 36% higher odds of VLBW compared to mothers with height greater than 150 cm (aOR: 1.36, 95% CI 1.29–1.43).

**Table 4.** Logistic regression of the selected characteristics with birth weight 1500-2499 g as outcome compared with normal birth weight - Model 2.

	LBW-Normal Birth Weight, (n= 111,266)		
	Prevalence of LBW	Odds Ratio	95% Conf. Interval
**Gender**			
Male	14.91	Ref	
Female	17.22	1.21 ***	1.15 to 1.26
**Birth order**			
One to three	15.88	Ref	
>4	16.53	0.86 ***	0.80 to 0.92
**Age of the mother**		
13–19 years	18.44	1.17 **	1.06 to 1.26
20–24 years	15.49	Ref	
>35 years	14.42	1.1	0.96 to 1.25
**Education**			
No education	18.18	1.08 *	1.02 to 1.15
Primary	17.88	1.16 **	1.08 to 1.25
Secondary	14.82	Ref	
**Social group**			
SC	17.94	1.07	0.99 to 1.16
ST	14.06	1.13 **	1.03 to 1.24
OBC	16.29	1.02	0.95 to 1.10
Others	15.32	Ref	
**Place of residence**			
Rural	16.18	1	0.94 to 1.06
Urban	15.40	Ref	
**Wealth index**			
Poorest/Poorer	17.33	1.11 ***	1.03 to 1.19
Middle	15.86	1.06	0.99 to 1.14
Rich/Richest	14.58	Ref	
**Geographic region**		
Northern states	17.92	1.09	0.98 to 1.20
Central states	18.32	0.97	0.88 to 1.06
Eastern states	15.05	0.75 ***	0.68 to 0.82
North Eastern States	9.83	0.61 ***	0.55 to 0.69
Western states	17.71	Ref	
Southern States	15.31	0.90 **	0.82 to 0.99
**Anaemia**			
Severe/Moderate	18.37	1.20 ***	1.13 to 1.26
Mild	14.79	0.95	0.89 to 1.01
Not anaemic	13.92	Ref	
**Tobacco Use**			
Users	14.04	0.96	0.87 to 1.06
Non-user	16.15	Ref	
**Alcohol drinking**		
Yes	16.00	0.94	0.76 to 1.16
No	13.39	Ref	
**Recommended ANC ^a^**		
No	13.32	Ref	
Yes	16.78	0.78 ***	0.73 to 0.83
**Multiple Pregnancy**		
Multiple	15.60	8.68 ***	7.05 to 10.68
Singleton	58.01	Ref	
**Height**			
<150 cm	18.28	1.36 ***	1.29 to 1.43
> 150 cm	14.66	Ref	

*p<.05 **p<.01 ***p<.001 ^a^ANC in first trimester, at least four antenatal visits, at least one tetanus toxoid (TT) injection and iron folic acid tablets or syrup taken for 100 or more days. SC- Scheduled Caste, ST- Scheduled Tribe, OBC- Other Backward Class, ANC- Antenatal Care, BMI- Body Mass Index

## Discussion

The programmatic factors included in the conceptual model as intermediate factors and the proximal factors were significant predictors of VLBW in India. Although the distal determinants such as the social and economic predictors were not independently associated with VLBW, they may directly influence the intermediate determinants and therefore influence the causal pathway. The study confirms that, VLBW is associated with several explanatory variables across different domains in the conceptual model, except the socio-economic determinants.

In our study girl children reported higher odds of presenting with LBW phonotypes as compared with boys. This is consistent with findings from other studies ^12, 13^. Male children in general have a tendency for higher birth weights and they are about 150 g heavier when compared to a female child and this difference in weight occurs often after 28 weeks of gestation ^14, 15^. Stunting in mothers is a significant predictor of both the LBW phenotypes. Comparison of our findings with those from other studies confirms that stunted mothers give birth to LBW child more often ^16, 17^ and it could be related to the growth restriction of the fetus in the smaller uterus of stunted mothers ^15^.

Our study showed association between birth order and LBW and age of the mother with both LBW and VLBW. Similar studies conducted elsewhere showed consistent findings related to the influence of maternal age and birth order on the birth weight of the child ^18– 20^. Maternal undernourishment and anaemia may have reflective effects on maternal weight gain and thereby birth weight of the child ^21, 22^. In our study, moderate to severe anaemia was associated with higher propensity for VLBW.

We demonstrate that educational status is an independent predictor of LBW. The odds of LBW were higher among mothers with “no education or primary level education” when compared with mothers with secondary level education. Educational level of the mother is one of the predictors of LBW in low-income countries ^12, 23, 24^. However, we could not determine consistent association between educational status of mother with VLBW. Similarly, our study did not establish relationship between child’s wealth quintile and VLBW. In contrast, a previous study from Brazil suggested an inverse association between family income with prevalence rates of VLBW ^25^. Further, belonging to a Scheduled Tribe increased the odds for LBW in our study. However, no evidence for increased risk of VLBW was detected for Scheduled Tribe population in our study and this is in contrast to the previous findings ^19, 26^.

Similar to the results of previous studies, our study demonstrates the association between lack of appropriate ANC and LBW phonotypes ^27– 29^. Evidence suggest that social determinants of health play a major role in access to health care, especially maternal health care in India. In India, the most pertinent social determinants influencing maternal health service utilization include socio-economic status, caste/ethnicity, education, gender, and religion ^30– 33^. Along with the above determinants, reports from NFHS-4 also points towards the influence of lack of husband’s participation in ANC and unintended pregnancies on lowering the odds for ANC utilization ^33^. Furthermore, the interaction between wealth and literacy is found to have a very strong role in maternal health care utilization indicators in India ^34^. The utilization of ANC and their determinants need to be explored in detail to recognize the barriers and opportunities to advance maternal health services in India.

Multiple pregnancies increased the odds of LBW and VLBW in our study. In India, there has been a progressive increase in availability of assisted reproductive technology (ART) services along with the advances in ART ^35– 37^. ART facilities like *in vitro* fertilization has raised the incidence of multiple pregnancy in the country due to preference for multiple embryo transfer, which increases the chance of a pregnancy ^38, 39^. Additionally, maternal parity is known to influence the incidence of LBW and VLBW infants ^40– 43^. 

Our study has some limitations. Firstly, birth weight was missing for more than 30,000 deliveries. The missing data were more from mothers who were from the marginalized communities. Mothers from lower socio-economic strata and disadvantaged population are known to have higher occurrence of LBW. Thus, our analysis could underestimate the various socio-economic factors associated with LBW in India. Secondly, information collected from the mothers on the antenatal and natal factors were from the past five years. Hence, the data quality is likely to be affected by recall bias.

## Conclusion

Despite having several common risk factors with the phenotypes of LBW and VLBW, the relationship is different in both the groups. For example, the social and economic determinants are unique to LBW. The VLBW is prominently associated with several genetic, nutritional, and demographic factors. The increasing trend in rate of multiple pregnancy and its association with VLBW poses a public health concern. Taken together, our results suggest that interventions geared towards improvements in antenatal care access, maternal health and nutritional status may reduce the number of VLBW infants in India. Interventions focused on reducing the number of VLBW infants can ultimately reduce infant mortality. Further, it may reduce the future burden of cardiovascular and metabolic disease conditions that are associated with VLBW.

## Data availability

Our study used data from the from individual recode file IAIR74DT, of the Demographic and Health Survey of India. The file mainly includes information on women in reproductive age group. Access to the data from DHS could be done using the link: https://www.dhsprogram.com/data/dataset/India_Standard-DHS_2015.cfm?flag=0 which needs prior registration of the research proposal. We registered our study with the DHS program and got access to the data. A guide for how to apply for dataset access is available at: https://dhsprogram.com/data/Access-Instructions.cfm. Supplementary Table 1 is available at 10.6084/m9.figshare.18393749). Supplementary Table 2 is available at 10.6084/m9.figshare.18393758)
