# Supplementary material for: Determinants of very low birth weight in India: The National Family Health Survey – 4
Source: Wellcome Open Res. 2022 May 17;7:20. Originally published 2022 Jan 24. [Version 2] doi: 10.12688/wellcomeopenres.17463.2 (PMC9171295; doi:10.12688/wellcomeopenres.17463.2)
Supplement: Peer review history

# Peer review: Reviewer response for version 2

**DOI:** 10.21956/wellcomeopenres.19825.r50617

**Published:** 2022-06-06

**Authors:** Malinee Laopaiboon

**Affiliations:** 1 Department of Epidemiology and Biostatistics, Faculty of Public Health, Khon Kaen University, Khon Kaen, Thailand

I am happy with the updated version and approve it.

Is the work clearly and accurately presented and does it cite the current literature?

Partly

If applicable, is the statistical analysis and its interpretation appropriate?

Not applicable

Are all the source data underlying the results available to ensure full reproducibility?

Partly

Is the study design appropriate and is the work technically sound?

Partly

Are the conclusions drawn adequately supported by the results?

Partly

Are sufficient details of methods and analysis provided to allow replication by others?

Partly

Reviewer Expertise:

NA

I confirm that I have read this submission and believe that I have an appropriate level of expertise to confirm that it is of an acceptable scientific standard.

# Peer review: Reviewer response for version 1

**DOI:** 10.21956/wellcomeopenres.19311.r48917

**Published:** 2022-03-11

**Authors:** Malinee Laopaiboon

**Affiliations:** 1 Department of Epidemiology and Biostatistics, Faculty of Public Health, Khon Kaen University, Khon Kaen, Thailand

1. This study was aimed to describe the determinants of very low birth weight (VLBW) in India based on the National Family Health Survey. However, the authors present the results of determinants of both VLBW (bw<1500 gm) and LBW (BW between 1500 and 2499 gm) infants. The results of LBW are out of this study's aim.
  I would suggest to analyse determinants of VLBW using infants with BW 1500-2499 gm to be another reference group compared to the results with the reference of normal BW infants. It may give more information to the readers.
2. We could call this data set a historical cohort. There was only 1.2 % of VLBW, which was rare. I would suggest using Poisson or negative binomial regression.
3. The authors present some errors,
  3.1 LBW in the methods of abstract section as BW <2500 gm.
  3.2 prevalence of categories of some factors should be in reverse categories as they are not consistent to adj OR, e.g. prevalence of multiple pregnancy (15.6%) versus single pregnancy (58.01%) while adjOR of multiple pregnancy was 8.68 for LBW, etc.

Is the work clearly and accurately presented and does it cite the current literature?

Partly

If applicable, is the statistical analysis and its interpretation appropriate?

Not applicable

Are all the source data underlying the results available to ensure full reproducibility?

Partly

Is the study design appropriate and is the work technically sound?

Partly

Are the conclusions drawn adequately supported by the results?

Partly

Are sufficient details of methods and analysis provided to allow replication by others?

Partly

Reviewer Expertise:

maternal and child health

I confirm that I have read this submission and believe that I have an appropriate level of expertise to state that I do not consider it to be of an acceptable scientific standard, for reasons outlined above.

# Response

**Published:** 2022-05-10

**Authors:** Panniyammakal Jeemon

**Affiliations:** Sree Chitra Tirunal Institute for Medical Sciences and Technology, Trivandrum, India

We thank the reviewer for their detailed comments. Please find below our response to the comments. 

**Comment: **This study was aimed to describe the determinants of very low birth weight (VLBW) in India based on the National Family Health Survey. However, the authors present the results of determinants of both VLBW (BW<1500 gm) and LBW (BW between 1500 and 2499 gm) infants. The results of LBW are out of this study's aim.

**Response**: Thank you, we have modified the objective statement of the study as;

 “We aim to describe the determinants of very low birth weight (VLBW) in India and compare it with the determinants of Low Birth Weight (LBW) based on the National Family Health Survey – 4 (NHFS-4)”

**Comment:** I would suggest analysing determinants of VLBW using infants with BW 1500-2499 gm to be another reference group compared to the results with the reference of normal BW infants. It may give more information to the readers.

**Response**: Thank you. As suggested, we analysed the determinants of VLBW using infants with BW 1500-2499 gm as another reference group and compared the results with the reference of normal BW infants. The results are included as supplementary table 4 ( 10.6084/m9.figshare.19556461) and supplementary table 5 ( 10.6084/m9.figshare.19556470).

 The results show that in the multivariable logistic regression model, mothers aged 13–19 years had higher odds for VLBW when compared with mothers aged 20–34 years (aOR: 1.35, 95% CI: 1.02–1.77). The odds of VLBW were 1.34 -times higher in mothers with severe or moderate anaemia *versus* non-anaemic mothers (aOR: 1.34, 95% CI: 1.10–1.62). Children from Eastern states had lower odds for VLBW (aOR: 0.62, 95% CI: 0.43–0.88) as compared with children from Western states. The odds of having VLBW was 2.20-times higher (aOR: 2.21, 95% CI: 1.53–3.17) among children of mothers with the multiple pregnancy *versus* singleton pregnancy. Preterm birth had higher odds of VLBW compared to term birth (aOR: 3.55, 95% CI: 2.90–4.33).

**Comment:** We could call this data set a historical cohort. There was only 1.2 % of VLBW, which was rare. I would suggest using Poisson or negative binomial regression.

**Response**: Thank you, we have done a Poisson Regression model and the results are added as supplementary table 3 ( 10.6084/m9.figshare.19556476). The Poisson model yielded similar results that of logistic regression model. Children who belonged to **scheduled tribe **had lower Incidence Rate Ratio (IRR) for VLBW versus children from other forward caste (IRR: 0.56, 95% CI: 0.45–0.69), (Logistic regression model: aOR: 0.82, 95% CI: 0.56 to 1.19). Children from **rich or richest wealth quintiles and middle wealth quintiles** had lower IRR of VLBW versus those from poorest/poorer wealth quintiles (IRR: 0.77, 95% CI: 0.65–0.91), (Logistic regression model: aOR: 0.89, 95% CI: 0.69 to 1.15).

**Comment: **The authors present some errors

 3.1 LBW in the methods of abstract section as BW <2500 gm.

**Response**: Thank you, we have corrected the LBW as Birth Weight 1500-2499 gm.

 3.2 Prevalence of categories of some factors should be in reverse categories as they are not consistent to adj OR, e.g., prevalence of multiple pregnancy (15.6%) versus single pregnancy (58.01%) while adjOR of multiple pregnancy was 8.68 for LBW, etc.

**Response**: Thank you, the prevalence of LBW in single pregnancies is 15.6% and the prevalence of LBW in multiple pregnancies is 58.8%. Therefore, the adjusted OR of multiple pregnancy is 8.68 for LBW. Thus, the results are consistent with the data.

# Peer review: Reviewer response for version 1

**DOI:** 10.21956/wellcomeopenres.19311.r48520

**Published:** 2022-03-07

**Authors:** Aditi Apte

**Affiliations:** 1 Vadu Rural Health Program, KEM Hospital Research Centre, Pune, Maharashtra, India

1. Title - Informative and balanced summary of what was done but does not mention about study design.
2. Introduction:
  Background & rationale - explains scientific background and rationale of study.
  Study design and setting - explained well.
  Selection criterion - well explained.
  Variables - clearly defined all outcomes, exposure, predictors but does not mentioned about potential confounders and effect modifiers. Also, number of USGs done in ANC period not taken into consideration.
3. Data sources - provided information about from where the data was extracted.
4. Bias - Not explained about efforts to address potential bias.
5. Study Size - explained well about sample size.
6. Statistical Methods - Multivariate analysis used but not mentioned how missing data was addressed.
7. Results - details about participants, descriptive and outcome data give. But, in the main results, the unadjusted estimates not given. Which confounders were adjusted for and why they were included is not mentioned in the study?
8. Discussion - Key results are summarised.
9. Limitations of study explained.
10. Generalisability – External validity of study results not discussed.

Above study has not taken into consideration one of the important social determinant i.e. about familial pressure on the woman for the want of male child which is responsible for higher birth order and subsequently LBW baby.

Is the work clearly and accurately presented and does it cite the current literature?

Yes

If applicable, is the statistical analysis and its interpretation appropriate?

I cannot comment. A qualified statistician is required.

Are all the source data underlying the results available to ensure full reproducibility?

Partly

Is the study design appropriate and is the work technically sound?

Yes

Are the conclusions drawn adequately supported by the results?

Yes

Are sufficient details of methods and analysis provided to allow replication by others?

Yes

Reviewer Expertise:

Maternal and child health

I confirm that I have read this submission and believe that I have an appropriate level of expertise to confirm that it is of an acceptable scientific standard.

# Response

**Published:** 2022-05-10

**Authors:** Panniyammakal Jeemon

**Affiliations:** Sree Chitra Tirunal Institute for Medical Sciences and Technology, Trivandrum, India

Thank you very much for approving our manuscript. Our responses to the comments are given below.

**Comment: **Title - Informative and balanced summary of what was done but does not mention about study design.

**Response**: The study title mentions NFHS-4, which indicates that it is a secondary data analysis of an existing national survey. 

**Comment:**

 Introduction: Variables - clearly defined all outcomes, exposure, predictors but does not mention about potential confounders and effect modifiers. Also, number of USGs done in ANC period not taken into consideration.

**Response**: Number of USGs done were classified into less than four and more than four and was used to calculate the variable ‘Antenatal care appropriate or not’. The confounders were adjusted in Model-1 and Model-2.

**Comment:** Data sources -provide information about from where the data was extracted.

**Response**: We have provided the source of NFHS-4 data.

**Comment: **Bias - Not explained about efforts to address potential bias.

**Response**: We used secondary data analysis for this study. National Family Health Survey data were collected by standardized techniques. This dataset was used widely in the Indian context. We have absolutely no control to address for any bias in the design or data collection. We have however identified potential confounding factors and included them in the multivariable model.

**Comment:** Study Size - explained well about sample size.

**Response**: Thank you.

**Comment: **Statistical Methods - Multivariate analysis used but not mentioned how missing data was addressed.

**Response**: The missing data in this study were considered missing and excluded from analysis. However, it was only a small fraction and may not have any significance in changing the effect estimates.

**Comment: **Results - details about participants, descriptive and outcome data given. But, in the main results, the unadjusted estimates not given. Which confounders were adjusted for and why they were included is not mentioned in the study?

**Response:** We have modified Table 3 and Table 4 to include unadjusted estimates. We used the following variables in the model as potential confounders, gender, birth order, mother’s age, education, social group, wealth index, geographic region, anaemia, tobacco use, alcohol use, ANC care, pregnancy type and height.

**Comment: **Generalisability – External validity of study results not discussed. Above study has not taken into consideration one of the important social determinants i.e., about familial pressure on the woman for the want of male child which is responsible for higher birth order and subsequently LBW baby.

**Response**: Thank you, NFHS-4 is a large database. It covers 28 states and 6 Union Territories. It is one of the large databases in India. Therefore, the findings are externally valid in the Indian population.  

 Our study shows that birth order is a determinant of LBW. Further data and investigation are required to establish relationship between familial pressure on the woman for the want of male child which leads to higher birth order and subsequently having LBW baby.
